# Associations of genotypes and haplotypes of *IL-17* with risk of gastric cancer in an eastern Chinese population

**DOI:** 10.18632/oncotarget.11616

**Published:** 2016-08-25

**Authors:** Fei Zhou, Li-Xin Qiu, Lei Cheng, Meng-Yun Wang, Jin Li, Meng-Hong Sun, Ya-Jun Yang, Jiu-Cun Wang, Li Jin, Ya-Nong Wang, Qing-Yi Wei

**Affiliations:** ^1^ Cancer Institute, Collaborative Innovation Center for Cancer Medicine, Fudan University Shanghai Cancer Center, Shanghai, China; ^2^ Department of Medical Oncology, Fudan University Shanghai Cancer Center, Department of Oncology, Shanghai Medical College, Fudan University, Shanghai, China; ^3^ Department of Oncology, Shanghai Jiaotong University Affiliated Shanghai First People's Hospital, Shanghai, China; ^4^ Department of Pathology, Fudan University Shanghai Cancer Center, Shanghai, China; ^5^ Ministry of Education Key Laboratory of Contemporary Anthropology, State Key Laboratory of Genetic Engineering, School of Life Sciences, Fudan University, Shanghai, China; ^6^ Fudan-Taizhou Institute of Health Sciences, Taizhou, Jiangsu, China; ^7^ Department of Gastric Cancer and Soft Tissue Sarcoma Surgery, Fudan University Shanghai Cancer Center, Shanghai, China; ^8^ Duke Cancer Institute, Duke University Medical Center, Durham, NC, USA

**Keywords:** interleukin-17, genetic variants, gastric cancer, susceptibility, molecular epidemiology

## Abstract

Interleukin-17 plays a crucial role in inflammation-related carcinogenesis. We hypothesize that genetic variants in *IL-17* are associated with gastric cancer (GCa) risk, and we genotyped five potentially functional single nucleotide polymorphisms (SNPs) (rs1974226 G > A, rs2275913 A > G, rs3819024 A > G, rs4711998 A > G, and rs8193036 C > T) of *IL-17* in 1121 GCa patients and 1216 cancer-free controls in an eastern Chinese population. Logistic regression analysis was used to calculate odds ratios (OR) and 95% confidence intervals (CI). Meta-analysis and genotype-mRNA expression correlation were performed to further validate positive associations. We found that an increased GCa risk was independently associated with rs1974226 (adjusted OR = 2.60, 95% CI = 1.27–5.32 for AA vs. GG + GA) and rs2275913 (adjusted OR = 1.33, 95% CI = 1.03–1.72 for GA + AA vs. GG), while a decreased GCa risk was independently associated with rs3819024 (adjusted OR = 0.72, 95% CI = 0.54–0.96 for GG vs. AA + AG). Additional meta-analyses confirmed the observed risk association with rs2275913. We also found that two *IL-17* haplotypes (G-G-G-A-C) and (A-G-G-A-C) (in the order of rs1974226, rs2275913, rs3819024, rs4711998 and rs8193036) were associated with a reduced GCa risk (adjusted OR = 0.64, 95% CI = 0.46–0.89 and adjusted OR = 0.38, 95% CI = 0.17–0.81, respectively). However, the expression Quantitative Trait Locus (eQTL) analysis for the genotype-phenotype correlation did not find mRNA expression changes associated with either the genotypes. In conclusions, genetic variants of *IL-17* are likely to be associated with risk of GCa, and additional larger studies with functional validation are needed to explore the molecular mechanisms underlying the observed associations.

## INTRODUCTION

Despite the advances in its diagnosis and therapy, gastric cancer (GCa) remains a great burden worldwide as the fifth most diagnosed cancer and the third most common cause for cancer-related deaths [1]. In China, there were an estimated 679,100 new cases and 498,000 deaths of GCa in 2015, ranking the second in both incidence and mortality of cancers [2]. Although the development of GCa is closely associated with personal lifestyles, such as smoking consumption, alcohol drinking and diet habit [3], the majority of GCa cases is attributable to the infection of *Helicobacter pylori (H. pylori)* and some to Epstein-Barr virus (EBV) [4, 5]. However, only a fraction of individuals who were exposed to these environmental factors eventually developed GCa, suggesting that genetic predisposition may have played an essential role in gastric carcinogenesis.

*Interleukin-17A* (*IL-17A*, also named *IL-17*), located on chromosome 6p12, encodes a glycoprotein of 155 amino acids [6]. IL-17 is the founding member of the cytokine family with six members (IL-17A, IL-17B, IL-17C, IL-17D, IL-17E, and IL-17F), it is mainly produced by the activated T cells, such as the T-helper 17 (Th17) cells, natural killer cells, mast cells and neutrophils, and it plays a crucial role in human immune system [7]. For example, IL-17 participates in mucosal immunity and fights against various bacteria, fungi, and EBV virus [8, 9]. *IL-17* transcripts have been observed to be higher in mucosal biopsies of *H. pylori*-infected gastritis patients, particularly for those with chronic inflammation [10]. IL-17 has been also reported to reduce *H. pylori* infection in mice contracted with *H. pylori*-related gastritis [11]. As infection-induced chronic gastritis is a major factor in histological development of GCa [12, 13], IL-17 has been speculated to play an important role in gastric pathogenesis.

In fact, IL-17 is also believed to be involved in both gastric carcinogenesis and progression, but the exact mechanisms remain to be elucidated. For example, it has been reported that IL-17, at the tumorigenesis stage, acts as a tumor suppressor involved in recruitment of T lymphocytes and dendritic cells (DCs), promotion of NK cell activity, activation of cytotoxic T lymphocytes (CTLs) and inhibition of T_reg_ repertoire in the tumor milieu [14, 15], whereas in the tumor progression stage, IL-17 inclines to have an oncogenic effect in inhibiting tumor cell apoptosis, impairing antitumor responses and promoting tumor angiogenesis, tumor metastasis and invasion [14, 15]. In GCa, some studies found that high expression levels of IL-17 predicted a better overall survival [16, 17], but others observed the opposite results [18–20].

Genetic variants, mostly represented by single nucleotide polymorphisms (SNPs), are believed to be involved in cancer susceptibility. Many studies of *IL-17* SNPs and susceptibility have focused on inflammation-related diseases [21–24], because IL-17 has been assumed as an important pro-inflammatory factor. Only a few studies have investigated the role of *IL-17* SNPs in susceptibility to cancers of the breast [25] and liver [26], and studies of GCa mainly focused on rs2275913 but generated conflicting conclusions [27–32].

To further test the hypothesis that there are associations between genetic variants of *IL-17* and GCa risk, we genotyped the five most representative, potentially functional SNPs of rs1974226, rs2275913, rs3819024, rs4711998, and rs8193036 in the gene in a hospital-based case-control study in an eastern Chinese population of 1126 cases and 1221 cancer-free controls. We not only analyzed the associations between these SNPs and GCa risk, but also evaluated the effects of the haplotypes on GCa risk as well as possible functional mechanisms that may underlie the observed positive associations.

## RESULTS

### Characteristics of the study population

The present study included 1121 cases and 1216 controls in the final analysis, including 305 (27.2%) gastric cardia adenocarcinoma (GCA) and 816 (72.8%) gastric non-cardia adenocarcinoma (GNCA) as described elsewhere [33]. As shown in Supplementary Table S1, there were no statistically significant differences in distribution of age and sex between cases and controls (*P* = 0.501 for age and *P* = 0.326 for sex). Compared with the cases, the controls had more smokers (49.1% versus 38.7%; *P* < 0.001) and drinkers (28.5% versus 23.7%; *P* = 0.008), but these variables were further adjusted for in the subsequent multivariate logistic regression analyses to evaluate the independent main effects of the selected SNPs under investigation.

### Associations between *IL-17* SNPs and GCa risk

Genotype frequency distributions of the five selected potentially functional SNPs are presented in Table 1. All these SNPs in the controls were in accordance with those expected from the Hardy-Weinberg Equilibrium (HWE, with *P* > 0.05 for all). The risk estimates of each SNP in different genetic models were adjusted by age, sex, smoking status and drinking status and other four SNPs.

**Table 1:** Logistic regression analysis of associations between selected SNPs of *IL-1*7 and gastric cancer risk in an eastern Chinese population

Variants	Genotype	Cases No. (%)	Controls No. (%)	*P*^a^	Crude OR (95% CI)	*P*^b^	Adjusted OR (95%CI)	*P*^b^
**All subjects, No.**		1121 (100.0)	1216 (100.0)					
***IL-17A* rs1974226 HWE_0.058**	GG	984 (87.8)	1049 (86.3)	**0.002^c^**	1.00		1.00	
	GA	110 (9.8)	156 (12.8)		**0.78 (0.60–0.97)**	**0.031**	0.78 (0.60–1.02)	0.074
	AA	27 (2.4)	11 (0.9)		**2.62 (1.29–5.30)**	**0.008**	**2.65 (1.28–5.47)**	**0.009**
**Trend test**					0.996		0.817	
**Dominant**	GA + AA	137 (12.2)	167 (13.7)	0.278^d^	0.88 (0.69–1.12)	0.279	0.91 (0.71–1.17)	0.466
**Recessive**	GG + GA	1094 (97.6)	1205 (99.1)		1.00		1.00	
	AA	27 (2.4)	11 (0.9)	0.004^e^	**2.70 (1.33–5.47)**	**0.006**	**2.60 (1.27–5.32)**	**0.009**
***IL-17A* rs2275913 HWE_0.160**	GG	330 (29.4)	389 (32.0)	0.361^c^	1.00		1.00	
	GA	560 (50.0)	576 (470.4		1.15 (0.95–1.38)	0.154	**1.34 (1.03–1.74)**	**0.032**
	AA	231 (20.6)	251 (20.6)		1.09 (0.86–1.37)	0.491	**1.55 (1.07–2.24)**	**0.021**
**Trend test**					0.392		**0.013**	
**Dominant**	GA + AA	791 (70.6)	827 (68.0)	0.182^d^	1.13 (0.95–1.34)	0.182	**1.33 (1.03–1.72)**	**0.031**
**Recessive**	GG + GA	890 (79.4)	965 (79.4)		1.00		1.00	
	AA	231 (20.6)	251 (20.6)	0.182^e^	1.00 (0.82–1.22)	0.983	1.24 (0.93–1.67)	0.145
***IL-17A* rs3819024 HWE_0.905**	AA	290 (25.9)	310 (25.5)	0.250^c^	1.00		1.00	
	AG	590 (52.6)	610 (50.2)		1.03 (0.85–1.26)	0.739	0.83 (0.63–1.11)	0.205
	GG	241 (21.5)	296 (24.3)		0.87 (0.69–1.10)	0.244	**0.63 (0.43–0.92)**	**0.017**
**Trend test**					0.265		**0.012**	
**Dominant**	AG + GG	831 (74.1)	906 (74.5)	0.835^d^	0.98 (0.81–1.18)	0.835	0.82 (0.63–1.08)	0.815
**Recessive**	AA + AG	880 (78.5)	920 (75.7)		1.00		1.00	
	GG	241 (21.5)	296 (24.3)	0.103^e^	0.85 (0.70–1.03)	1.103	**0.72 (0.54–0.96)**	**0.026**
***IL-17A* rs4711998 HWE_0.764**	AA	571 (50.9)	644 (53.0)	0.420^c^	1.00		1.00	
	AG	471 (42.0)	479 (39.4)		1.11 (0.94–1.31)	0.233	1.08 (0.92–1.30)	0.393
	GG	79 (7.1)	93 (7.7)		0.96 (0.70–1.32)	0.794	0.96 (0.68–1.35)	0.814
**Trend test**					0.584		0.661	
**Dominant**	AG + GG	550 (49.1)	572 (47.0)	0.328^d^	1.08 (0.92–1.28)	0.328	1.09 (0.92–1.30)	0.305
**Recessive**	AA + AG	1042 (92.9)	1123 (92.3)		1.00		1.00	
	GG	79 (7.1)	93 (7.7)	0.579^e^	0.92 (0.67–1.25)	0.579	0.90 (0.65–1.25)	0.539
***IL-17A* rs8193036 HWE_0.576**	CC	558 (49.8)	625 (51.4)	0.272^c^	1.00		1.00	
	CT	482 (43.0)	488 (40.1)		1.11 (0.93–1.31)	0.244	1.03 (0.85–1.26)	0.736
	TT	81 (7.2)	103 (8.5)		0.88 (0.64–1.20)	0.426	0.85 (0.59–1.21)	0.361
**Trend test**					0.886		0.839	
**Dominant**	CT + TT	563 (50.2)	591 (48.6)	0.434^d^	1.07 (0.91–1.26)	0.434	1.07 (0.89–1.27)	0.487
**Recessive**	CC + CT	1040 (92.8)	1113 (91.5)		1.00		1.00	
	TT	81 (7.2)	103 (8.5)	0.264^e^	0.84 (0.62–1.14)	0.265	0.81 (0.59–1.12)	0.201

Abbreviation: SNP, single nucleotide polymorphism; OR, odds ratio; CI, confidence interval; HWE, Hardy Weinberg Equilibrium.

a Chi square test for genotype distributions between cases and controls.

b Adjustment without (crude) and with age, sex, smoking and drinking status and all these five SNPs in logistic regression models.

c For additive genetic models.

d For dominant genetic models.

e For recessive genetic models.

From the results and trend tests presented in Table 1, the number of the rs2275913 A allele was significantly associated with an increased GCa risk (*P* = 0.013) but the number of the rs3819024 G allele was significantly associated with a decreased GC risk (*P* = 0.012) in an allele dose-response manner. The number of variant alleles of other three SNPs did not have such an obvious tend in association with GCa risk.

In a recessive genetic model with multivariate adjustment (Table 1), GCa risk was independently associated with the rs1974226 AA genotype (adjusted OR = 2.60, 95% CI = 1.27–5.32 for AA vs. GG + GA) and the rs3819024 GG genotype (adjusted OR = 0.72, 95% CI = 0.54–0.96 for GG vs. AA + AG), while the rs2275913 GA + AA genotypes were independently associated with an increased risk in a dominant genetic model (adjusted OR = 1.33, 95% CI = 1.03–1.72 for GA + AA vs. GG). However, these risks were not observed for other two SNPs (Table 1).

### SNP-SNP and SNP-environment interactions

As shown in Table 2, the logistic regression analyses identified a significant interaction between rs2275913 and rs3819024 SNPs (*P* = 0.009). However, we did not find evidence for any other significant SNP-SNP or SNP-environment interactions.

**Table 2:** SNP-SNP and SNP-environment interactions (*P* value) between the three positive associated SNPs in *IL-1*7 (logistic regression)

	rs1974226	rs2275913	rs3819024	Smoking	Drinking
**s1974226**					
**rs2275913**	0.641				
**rs3819024**	0.398	**0.009**			
**Smoking**	0.960	0.474	0.986		
**Drinking**	0.390	0.943	0.112	0.244	

### Meta-analysis for the association between rs2275913 and GCa risk

**Figure 1:**
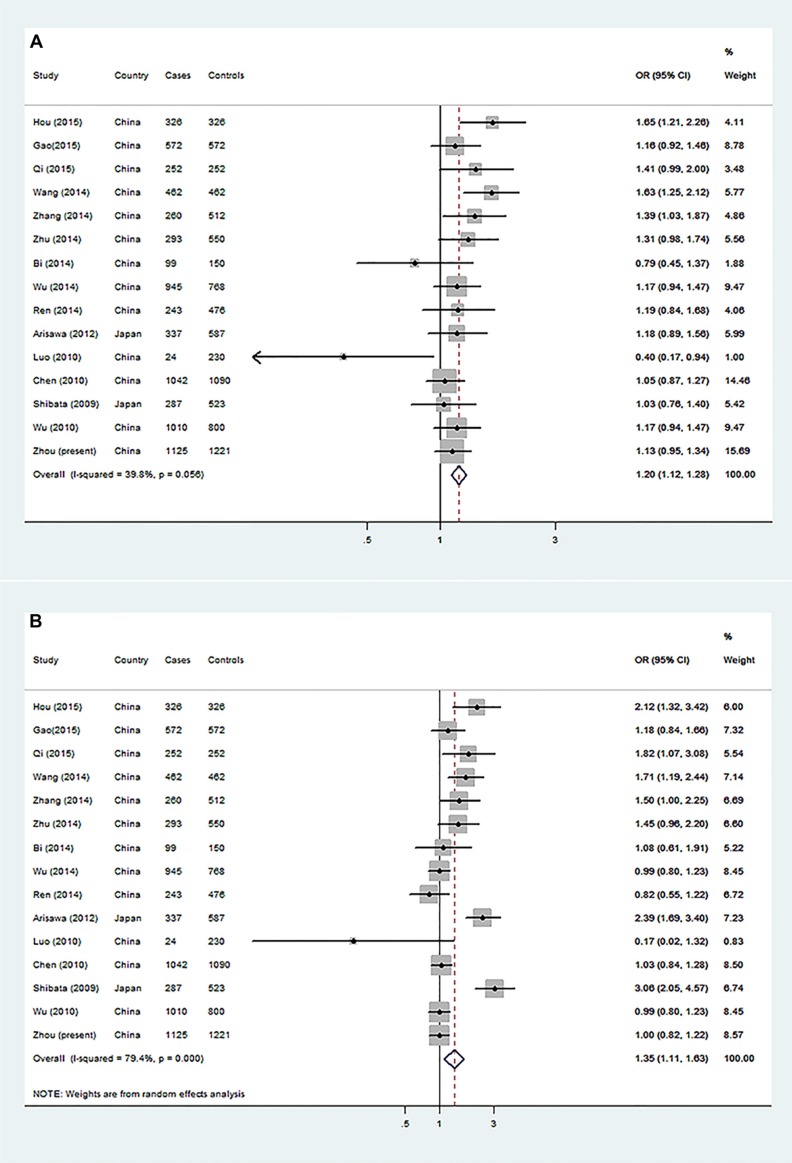
Forest plot of gastric cancer risk associated with *IL-17* rs2275913 from a meta-analysis of 15 case-control studies The OR and 95% CI of each study are plotted with a box and a horizontal line. Quadrangles represent pooled ORs and 95% CI. (**A**) GA + AA vs. GG in a dominant genetic model and (**B**) AA vs. GG + GA in a recessive genetic model.

Because the rs2275913 SNP has been mostly investigated for its association with GCa risk in published studies, we were able to conduct a mini-meta analysis with all the published data available for Asians. By including our new dataset, the meta-analysis consisted of a total of 7277 cases and 8519 controls. We found that the A allele was significantly associated with an increased GCa risk in either dominant (OR = 1.20, 95% CI = 1.12–1.28 for GA + AA vs. GG) or recessive (OR=1.35, 95% CI = 1.11–1.63, for AA vs. GA + GG) genetic models (Figure 1A–1B). Furthermore, the shapes of Begg's and Egger's plots showed no significance publication bias for this mini-meta analysis (Supplementary Figure S1A–S1D). Because there were few studies about the other SNPs in *IL-17*, we were not able to perform the meta-analyses accordingly.

### Stratification analysis

We further performed a stratification analysis by dichotomized variables of age, sex, smoking status and drinking status for the three significantly independent SNPs (i.e., rs1974226, rs2259713 and rs3819024) (Table 3). A significantly increased GCa risk was associated with the *IL17* rs1974226 AA variant genotype in subgroups of older age (adjusted OR = 2.99, 95% CI = 1.12–7.95), males (adjusted OR = 2.56, 95% CI = 1.11–5.90), never-smokers (adjusted OR = 2.72, 95% CI = 1.07–6.96), never drinkers (adjusted OR = 2.87, 95% CI = 1.20–6.91). We also found that the rs3819024 GG variant genotype was associated with a decreased GCa risk in never-smoker subgroup (adjusted OR = 0.76, 95% CI = 0.58–0.98). However, the homogeneity test did not show that there were differences in stratum ORs for these dichotomized variables. These significant risk associations were not found for other SNPs in the stratification analysis (data not shown).

**Table 3:** Stratification analysis for associations of *IL1*7 rs1974226, rs2275913 and rs3819024 with gastric cancer risk in an eastern Chinese population

Variables	*IL17* s1974226 (cases/controls)						*IL17* rs2275913 (cases/controls)					*IL17* rs3819024 (cases/controls)				
	GG + GA	AA	Adjusted OR (95% CI)	*P*^a^	*P*^b^	GG + GA	AA	Adjusted OR (95% CI)	*P*^a^	*P*^b^	AA + AG	GG	Adjusted OR (95% CI)	*P*^a^	*P*^b^
**Age**															
** ≤ 59**	563/607	12/5	2.50 (0.87–7.15)	0.089	0.807	453/487	122/125	1.05 (0.79–1.39)	0.752	0.590	452/472	123/140	0.91 (0.69–1.20)	0.501	0.751
** > 59**	531/589	15/6	**2.99 (1.12–7.95)**	**0.028**		437/478	109/126	0.94 (0.70–1.26)	0.650		428/448	118/156	0.81 (0.61–1.07)	0.138	
**Sex**															
** Females**	316/376	7/3	3.03 (0.76–12.09)	0.117	0.841	259/296	64/77	0.93 (0.64–1.35)	0.710	0.748	257/287	166/86	0.85 (0.59–1.23)	0.387	0.957
** Males**	778/835	20/8	**2.56 (1.11–5.90)**	**0.028**		631/669	167/174	1.00 (0.79–1.27)	0.984		623/633	175/210	0.84 (0.67–1.06)	0.150	
**Smoking status**															
** Never**	669/613	18/6	**2.72 (1.07–6.96)**	**0.036**		549/484	138/135	0.90 (0.68–1.17)	0.424	0.278	548/464	139/155	**0.76 (0.58–0.98)**	**0.037**	0.169
** Ever**	425/592	9/5	2.77 (0.91–8.41)	0.072	0.980	341/481	93/116	1.13 (0.83–1.53)	0.451		332/456	102/141	1.00 (0.75–1.34)	0.879	
**Drinking status**															
** Never**	835/862	20/7	**2.87 (1.20–6.91)**	**0.018**		670/685	185/184	1.03 (0.82–1.30)	0.802	0.483	666/660	189/209	0.91 (0.72–1.14)	0.391	0.340
** Ever**	259/343	7/4	2.30 (0.66–7.96)	0.190	0.776	220/280	46/67	0.87 (0.58–1.32)	0.516		214/260	52/87	0.73 (0.49–1.07)	0.106	

a Obtained in logistic regression models without (crude) and with adjustment for age, sex, smoking and drinking status. OR, odds ratio; CI, confidence interval.

b *P* for homogeneity test using the χ^2^ test.

### Association between haplotypes of *IL-17* SNPs and GCa risk

We observed an increased GCa risk associated with rs1974226 in a recessive genetic model and rs2275913 in a dominant genetic model, while the rs3819024 was associated with a decreased GCa risk in a recessive genetic model. To account for all five SNPs in the same gene, we performed a haplotype analysis to explore their combined genetic effects. As shown in Table 4, we found that haplotypes G-G-G-A-C and A-G-G-A-C (in the order of rs1974226, rs2275913, rs3819024, rs4711998 and rs8193036) were significantly associated with a decreased GCa risk (adjusted OR = 0.64, 95% CI = 0.46–0.89 and adjusted OR = 0.38, 95% CI = 0.17–0.81, respectively), compared with the most frequent haplotype G-A-G-A-C.

**Table 4:** Haplotype analysis for association between *IL-17* and GCa risk in an eastern Chinese population

Haplotypes*	Haplotype frequency				Crude OR (95% CI)	*P*	Adjusted OR (95% CI)	*P*^a^
	Case (*N* = 2242)		Control (*N* = 2432)					
	*N*	%	*N*	%				
**G-A-G-A-C**	758	33.81	779	32.03	1.00		1.00	
**G-G-A-A-C**	560	24.98	618	25.41	0.93 (0.80–1.08)	0.358	0.93 (0.80–1.09)	0.357
**G-G-A-G-T**	342	15.25	352	14.47	1.00 (0.84–1.20)	0.987	0.99 (0.83–1.19)	0.920
**G-G-A-A-C**	190	8.47	206	8.47	0.95 (0.76–1.18)	0.635	0.96 (0.77–1.20)	0.691
**G-G-A-G-C**	158	7.05	160	6.58	0.95 (0.76–1.18)	0.905	1.02 (0.80–1.30)	0.871
**G-G-G-A-C**	64	2.85	102	4.19	**0.65 (0.46–0.90)**	**0.009**	**0.64 (0.46–0.89)**	**0.008**
**A-G-A-A-C**	55	2.45	64	2.63	0.88 (0.61–1.28)	0.515	0.87 (0.60–1.27)	0.466
**A-G-A-G-C**	42	1.87	45	1.97	0.96 (0.62–1.48)	0.85	1.00 (0.64–1.54)	0.988
**G-A-A-A-C**	33	1.47	48	1.85	0.71 (0.45–1.11)	0.134	0.71 (0.45–1.12)	0.139
**A-G-A-A-T**	23	1.03	18	1.03	1.31 (0.70–2.45)	0.393	1.32 (0.70–2.48)	0.396
**A-G-G-A-C**	9	0.4	25	0.74	**0.37 (0.17–0.80)**	**0.011**	**0.38 (0.17–0.81)**	**0.013**
**A-G-A-G-T**	8	0.36	15	0.62	0.55 (0.23–1.30)	0.173	0.63 (0.26–1.51)	0.298

* The alleles in the haplotype were ranked in the SNP order of rs1974226G/A, rs2275913G/A, rs3819024A/G, rs4711998A/G, and rs8193036C/T. OR, odds ratio; CI, confidence interval.

a Obtained in logistic regression models with adjustment for age, sex, smoking status and drinking status.

### Functional exploration with the expression Quantitative Trait Locus (eQTL) analysis by *IL-17* genotypes in lymphoblastoid cell lines

To explore the mechanisms underlying the observed associations, we further performed genotype-phenotype correlation analysis by using mRNA expression data of the lymphoblastoid cell lines derived from 79 unrelated Chinese people available in the HapMap 3 database. As shown in Supplementary Figure S2A–S2E, for each of the five SNPs (rs1974226, rs2275913, rs3819024, rs4711998 and rs8193036), none of their mRNA expression levels of *IL-17* was correlated with the variant allele (*P*_trend_ = 0.491, 0.441, 0.848, 0.680, and 0.667, respectively), compared with their common alleles, respectively.

## DISCUSSION

Mounting evidence has demonstrated that inflammation in tumor microenvironment has a role in promoting cancer cell proliferation and migration, while initiating and maintaining protective antitumor immunity, but the beneficial or detrimental effects of inflammation in terms of tumor development depend on the nature of inflammatory response and the tissue specificity [34, 35]. IL-17 is an effector cytokine that is produced by cells of both the innate and adaptive immune systems, constructing a bridge in the inflammatory reactions [36]. IL-17 was first discovered for its effects on synoviocytes from patients with rheumatoid arthritis [37], and later it was found to be involved in eliminating pathogens like gram-positive *Propionibacterium acne*, gram-negative *Citrobacter rodentium*, and fungi such as *Candida albicans* [38]. With the discovery of Th17 cell, IL-17 was identified as an important component of the tumor-associated immune response in the tumor microenvironment, but its exact role in carcinogenesis is still not fully understood.

To our best knowledge, the present study is the first to explore the role of all potentially functional SNPs of *IL-17*, particularly rs1974226 in the 3′UTR region with a potential influence on miRNA binding, in cancer risk, because the *IL-17* rs1974226 AA variant genotype was associated with an increased GCa risk. Although no studies have reported an association between rs1974226 and cancer risk, two previous studies have investigated associations between rs1974226 and inflammation-related diseases. In a study of ulcerative colitis, the rs1974226 G allele in a haplotype was found to be protective for Crohn's disease [22], suggesting that the A allele was relatively a risk factor, which is consistent with the findings in the present study.

Many studied investigated *IL-17* rs2275913 and GCa cancer risk but had generated conflicting conclusions, some reporting no association [27–29] and some demonstrating an association with GCa risk [30–32]. In the present study, we found that the *IL-17* rs2275913 A allele was associated with an increased GCa risk. Considering the varying ethnic difference in cancer susceptibility, we did a meta-analysis with previously published studies in Asian populations. Overall, we confirmed an association between the rs2275913 A allele and GCa risk in both dominant and recessive genetic models, which did not agree with some previously published meta-analyses [39–41], likely due to the fact that the present study added a large sample size to the present meta-analysis, resulting in an increased statistical power.

In addition, we also found that the GG genotype of rs3819024 was associated with a decreased GCa risk. There were only two published studies about the association between rs3819024 and cancer risks. One breast cancer study found that the A allele was associated with a reduced risk, if women consumed a high folate diet [25], while the other GCa study identified the rs3819024 G allele to be marginally associated with a decreased GCa risk in a recessive genetic model [29], a finding consistent with the present study. The opposite effect of the A allele in breast cancer and GCa may be disease-specific, but this speculation needs further validation by additional studies.

There was only one publication about the rs4711998 SNP and gastrointestinal cancer risk, suggesting that the A allele tended to be associated a non-significantly decreased hepatocellular carcinoma risk [26]. A breast cancer study revealed that carriers with the rs8193036 CC genotype had higher breast cancer mortality [25], but no published studies have evaluated rs8193036 for its association with cancer risk.

We assessed SNP-SNP and SNP-environment interactions for these three positive associated SNPs. We found that there was a SNP-SNP interaction between rs2275913 and rs3819024, suggesting that these two SNPs had a joint effect in modulating the risk of GCa.

In the haplotype analysis, we found that haplotypes G-G-G-A-C and A-G-G-A-C (alleles in the order of rs1974226G/A, rs2275913G/A, rs3819024A/G, rs4711998A/G, and rs8193036C/T) were associated with a decreased GCa risk. Except for the first allele in the order of variant alleles, the other four alleles in these two haplotypes were identical. In the trend analysis for the four SNPs, the rs2275913 G allele and rs3819024 G alleles were significantly associated with a decreased GCa risk, while the rs4711998A and rs8193036C alleles were insignificantly associated with a reduced GCa risk. The trend analysis results for these SNPs almost fit with their roles in the haplotypes. For rs1974226, although the AA genotype was obviously associated with a decreased GCa risk, it was not statistically significant in the trend analysis for the A allele, because it fit well with a recessive genetic model.

Since three (rs1974226, rs2275913 and rs3819024) of the five SNPs were found to be independently associated with GCa risk in the present study, we explored their molecular mechanism underlying the observed associations. Unfortunately, mRNA expression data from 79 unrelated Chinese people available in the HapMap 3 did not show the genotype-phenotype correlation. It is likely that these common variants may have limited and weak influence on mRNA expression not detectable in a sample of 79 individuals. Among the three positive SNPs, two are located at the 5′ near gene and one is located at the microRNA binding site. Additional mechanistic studies are warranted to explore their functional relevance to the observed associations.

It was regretful that we did not find the correlation between IL-17 mRNA expression and genotypes of the SNPs. Because these SNPs were chosen as a tag of the gene, other untagged SNPs of the genes may be functional and could have been missed by this study. Future fine mapping of the gene is necessary to unravel such functional SNPs.

There were some limitations in the present study. First, although age, sex, smoking history, and drinking history were considered, there were some factors that may have contributed to GCa risk but were not included in the analyses, e.g. H. pylori infection status, family history, diet habit, nutrition status and socioeconomic status. Second, the numbers of SNPs genotyped in the present study were limited, and some rare potentially functional variants in the genes might have been missed, which may need additional sequencing. Third, we have no plasma and tissue samples available to do the further functional analysis. Therefore, the results of the present study should be interpreted with caution.

In conclusions, in the present study, we investigated associations between five potentially functional SNPs of the *IL-17* gene and GCa risk in an eastern Chinese population. There are some published studies that have dealt with the role of SNPs in the *IL-17* gene in GCa risk. One study included more SNPs but a small sample of only about 200 cases [29]. Another study included a large sample of more than 1,000 cases, but only studied one SNP in the *IL-17* gene [27]. Most published studies included just one to three SNPs and usually with small samples of about 200–500 cases [30, 42–45]. The present study not only analyzed all the potentially functional SNPs located at the transcription factor binding site (TFBS) in the putative promoter region or at the microRNA (miRNA) binding site of *IL-17* in a large sample size of more than 1,000 cases and 1,000 controls, and but also did haplotype analysis. We found that the rs1974226 AA genotype was independently associated with an increased GCa risk in a recessive genetic model, but that the rs2275913 GA/AA genotypes were independently associated with an increased GCa risks in a dominant model, consistent with the findings from further meta-analysis of all published studies of this SNP. We also found that the rs3819024 GG genotype was independently associated with a decreased GCa risk in a recessive model. There was an SNP-SNP interaction between rs2275913 and rs3819024. We next found that haplotypes G-G-G-A-C and A-G-G-A-C (in the order of rs1974226, rs2275913, rs3819024, rs4711998 and rs8193036) were independently associated with a decreased GCa risk, compared with the most common haplotype G-A-G-A-C, consistent with the results of single locus analysis. Further genotype-mRNA expression correlation analysis for all these five SNPs, however, failed to find evidence for functional relevance for any of the variant genotypes. Larger, more stringently designed studies are needed to validate our findings.

## MATERIALS AND METHODS

### Study subjects

The final analysis consisted of 1,121 unrelated Han Chinese patients with newly diagnosed and histopathologically confirmed primary gastric adenocarcinoma from an ongoing molecular epidemiology study at Fudan University Shanghai Cancer Center (FUSCC) between 2009 and 2011 [33]. All patients came from eastern China, including Shanghai, Jiangsu, Zhenjiang and the surrounding area. Besides, 1,216 Han ethnic cancer-free controls, recruiting from Taizhou Longitudinal Study (TZL) in eastern China at the same period, were frequency matched to the cases on age (± 5 years) and sex (Supplementary Table S1). Blood samples of these GCa patients and cancer-free controls came from the tissue bank of FUSCC and the TZL study, respectively. All subjects in the study signed a written informed consent for donating their biological samples to the FUSCC tissue bank and TZL study for scientific research. This study was approved by the Institutional Review Board of FUSCC.

### SNP selection and genotyping

**Figure 2:**
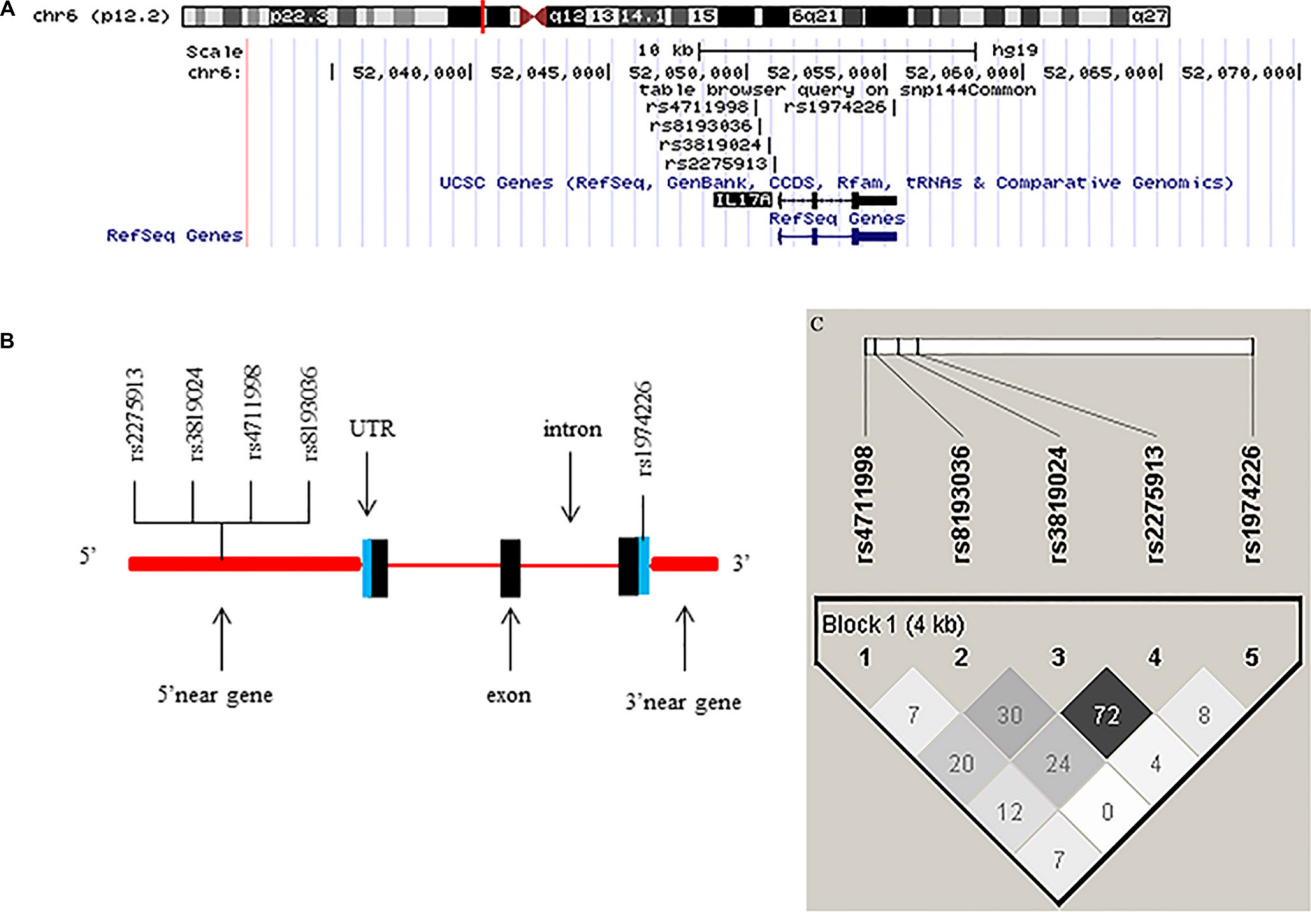
Chromosome and gene maps and locations of the potentially functional polymorphisms in the *IL-17* gene as predicted by SNPinfo (**A**) The chromosome structure showing the location of the IL-17 gene from UCSC browser (NCBI137/hg19); (**B**) The IL-17 gene structure showing the locations of the potential functional SNPs selected and studied in the present study; (**C**) Linkage disequilibrium (LD) blocks of *IL-17* genes. The value within each diamond represents the pairwise correlation between SNPs (measured as *r*^2^) defined by the upper left and the upper right sides of the diamond.

The SNPs to be genotyped were selected from the NCBI dbSNP database (http://www.ncbi.nlm.nih.gov/projects/SNP) and SNPinfo (http://snpinfo.niehs.nih.gov/snpfunc.htm) using following criteria: (1) located at the TFBS in the putative promoter region; (2) located at the miRNA binding site; (3) the minor allele frequency (MAF) of at least 5% in Chinese populations; (4) with a low linkage disequilibrium (LD) with other SNPs using an r^2^ threshold of < 0.8 as the cut-off value for each other, and (5) not included in the published genome-wide association studies (GWASs) (Table 5). As a result, only five SNPs in *IL-17* satisfied the above criteria: rs1974226 located in the 3′UTR region, which may affect the miRNA binding site activity, and the other four SNPs (rs2275913, rs3819024, rs4711998 and rs8193036) located in the 5′ near gene region, which may affect the TFBS activity (Table 5 and Figure 2A and 2B). The LD block of the five SNPs was shown in Figure 2C, suggesting that there was no LD between these SNPs. Taqman real-time PCR method was employed to genotype these five selected SNPs with genomic DNA extracted from the blood samples, and genotyping was performed as previously described [46], with a successful call rate of more than 99.5%. The discrepancy rate in 10% of the samples for duplication was less than 0.1%, and some samples were also randomly selected for direct sequencing to confirm the observed genotypes.

**Table 5:** The selected, potentially functional SNPs as predicted by SNPinfo software

Gene	SNP rs no.	Chromosome no.	Gene region	Functional prediction	Major/Minor allele	Minor frequency in Asians*	Minor frequency in CHB*
***IL-17***	rs1974226	6	3′UTR	miRNA binding site	G/A	0.060	0.072
***IL-17***	rs2275913	6	5′ near gene	TFBS	G/A	0.302	0.506
***IL-17***	rs3819024	6	5′ near gene	TFBS	A/G	0.476	0.518
***IL-17***	rs4711998	6	5′ near gene	TFBS	A/G	0.212	0.208
***IL-17***	rs8193036	6	5′ near gene	TFBS	C/T	0.338	0.286

TFBS: transcription factor binding sites; UTR: untranslated region; CHB: Chinese Beijing, Han.

* Data from HapMap phase 3 in 137.

### Genotype and mRNA expression data of lymphoblastoid cell lines from the HapMap3 database

We also used additional genotype data of *IL-17* (http://hapmap.ncbi.nlm.nih.gov/downloads/genotypes/2010-05_phaseIII/) and mRNA expression data that are available online (http://www.ebi.ac.uk/arrayexpress/experiments/E-MTAB-264/) for the genotype-phenotype correlation analysis. The genotyping data were from the HapMap phase 3 release 3 dataset consisting of about 1.6 million SNP genotypes of 692 individuals from 11 populations [47]. The mRNA expression data together with genotypes were derived from EBV-transformed B lymphoblastoid cell lines obtained from 726 individuals from 8 global populations from the HapMap3 Project [48].

### Meta-analysis of the association between *IL-17* variants and GCa risk using published studies

Briefly, the keywords search was performed with or without the Medical Subject Headings (MeSH) terms for: ‘interleukin-17 or IL-17’, ‘variant or polymorphism’, and ‘gastric or stomach’ and ‘cancer or neoplasm or malignancy’. Studies included in the meta-analysis must meet the following inclusion criteria: case-control designed studies; studies evaluating the association of *IL-17* variants with GCa risk; studies with sufficient data for estimating genotype frequency. The main reasons for exclusion were abstract, reviews, duplicate data, of family cancers, not in English or Chinese, and not about GCa. Relevant studies were searched from PubMed and Chinese National Knowledge Infrastructure (CNKI) databases (Last Updated: March 4, 2016). The pooled ORs and 95% CIs were calculated under dominant and recessive models. χ^2^-based Q-test was used to check heterogeneity among the studies. Either the fixed-effects (the Mantel-Haenszel method) or random-effects (the DerSimonian and Laird method) model was chosen to calculate the pooled OR estimates according to the heterogeneity of study populations included in the meta-analysis. The Begg's and Egger's linear regressions were used to test the potential publication bias. Sensitivity analysis was also performed to assess the stability of the meta-analysis. All statistical tests were performed with STATA (version 12.0; Stata Corporation, College Station, TX).

### Statistical analysis

Differences of categorical variables distribution were calculated by Pearson's χ^2^ test between cases and controls, and differences in continuous variables between cases and controls were evaluated by Student's *t* test. The HWE was tested by a goodness-of-fit χ^2^ test in the control group. GCa risks in both overall and stratified subgroup analyses were evaluated by ORs and 95% CIs with both univariate and multivariate logistic regression analyses. mRNA expression levels between samples of different genotypes were compared by the general linear regression analysis. Based on the observed genotypes, PROC HAPLOTYPE was used to generate individual haplotypes and frequencies to calculate ORs and 95% CIs associated with GCa risk in the logistic regression analysis. All tests were two-sided using the Statistical Analysis Software (SAS, v.9.4, SAS Institute, Cary, NC), and *P* < 0.05 was considered statistically significant.

## SUPPLEMENTARY MATERIALS



## Abbreviations

CI: confidence interval
eQTL: expression Quantitative Trait Locus
Epstein-Barr virus: EBV
GCa: gastric cancer
GCA: gastric cardia adenocarcinoma
GNCA: Gastric non-cardia adenocarcinoma
GWASs: genome-wide association studies
HWE: Hardy Weinberg equilibrium
H. pylori: Helicobacter pylori
IL-17: interleukin-17
LD: linkage disequilibrium
MAF: minor allele frequency
microRNA: miRNA
OR: odds ratios
SNP: single nucleotide polymorphism
TFBS: transcription factor binding site
T-helper 17: Th17.
