# Supplementary material for: Associations of genotypes and haplotypes of IL-17 with risk of gastric cancer in an eastern Chinese population
Source: Oncotarget. 2016 Aug 25;7(50):82384–95. doi: 10.18632/oncotarget.11616 (PMC5347698; doi:10.18632/oncotarget.11616)
Supplement: Supplementary file 1 [file oncotarget-07-82384-s001.pdf]

Associations of genotypes and haplotypes of *IL-17* with risk of gastric cancer in an eastern Chinese population

Supplementary Materials

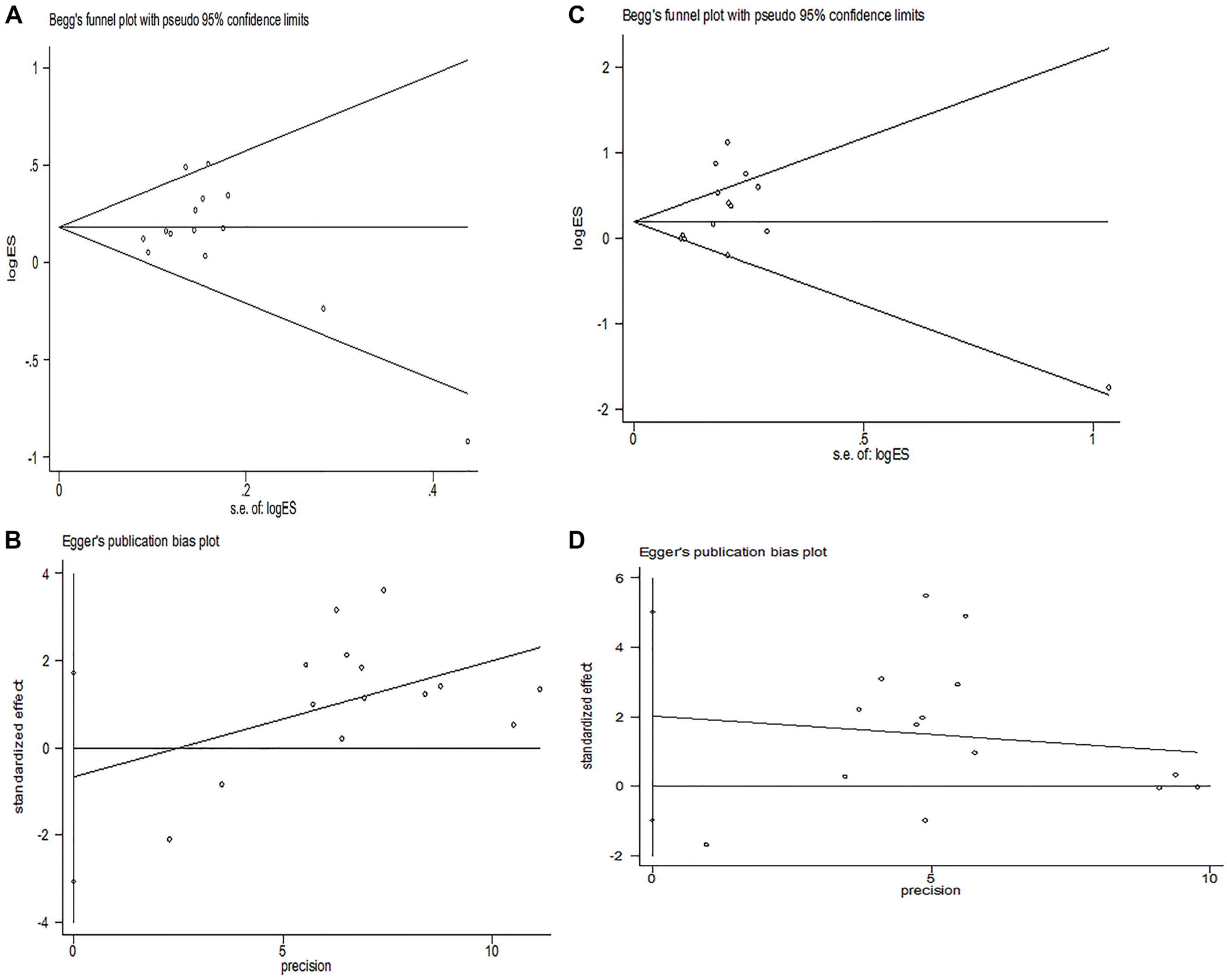

Supplementary Figure S1: Begg's and Egger's plot evaluating publication bias from the eligible published studies. (**A**) Begg's plot for rs2275913 dominant model ($P = 0.805$); (**B**) Egger's plot for rs2275913 dominant model ($P = 0.553$); (**C**) Begg's plot for rs2275913 recessive model ($P = 0.299$); (**D**) Egger's plot for rs2275913 recessive model ($P = 0.170$).

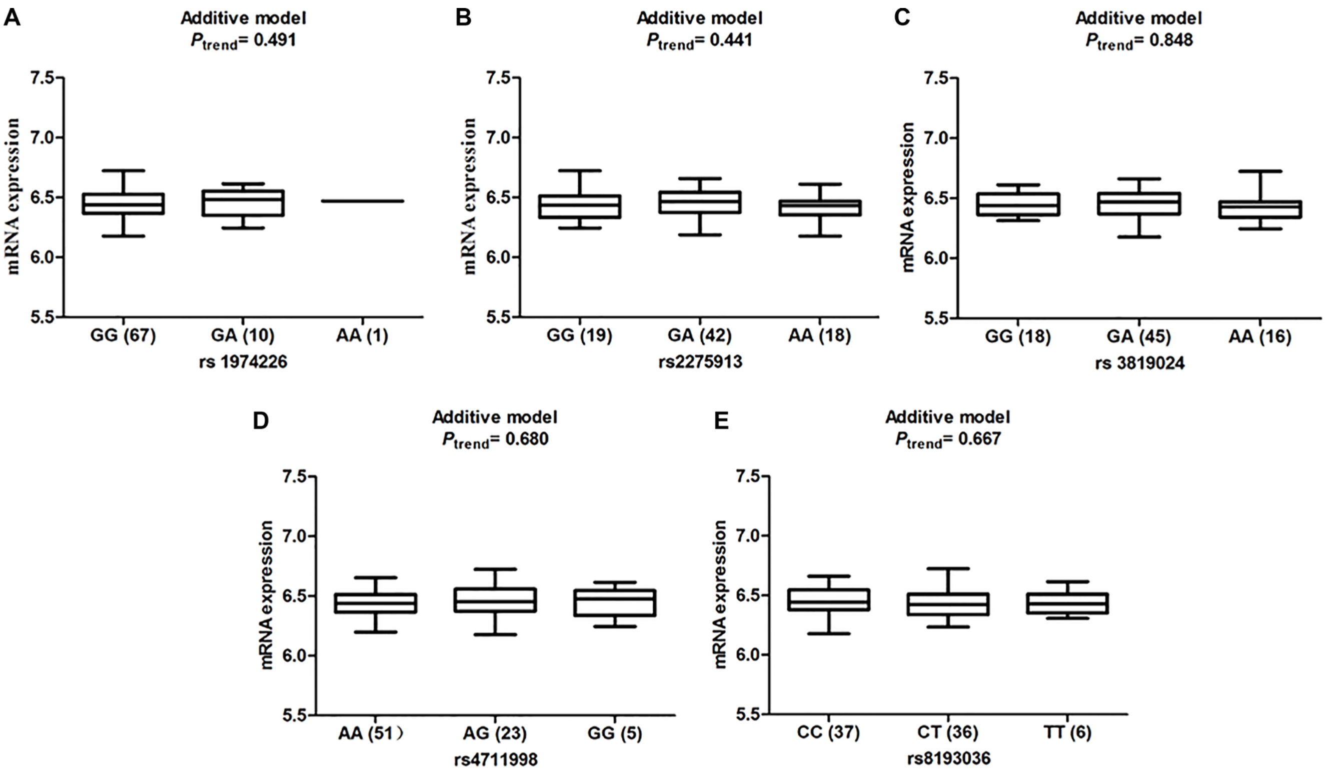

Supplementary Figure S2: eQTL analysis of mRNA expression for genotype-phenotype correlation from EBV-transformed B lymphoblastoid cell lines of 79 unrelated Chinese people included in HapMap 3 database. (**A**) for rs1974226; (**B**) for rs2275913; (**C**) for rs3819024; (**D**) for rs4711998; (**E**) for rs8193036.

Supplementary Table S1: Demographic characteristics of gastric cancer cases and cancer-free controls in an eastern Chinese population

Variables	Cases<br>*N* (%)	Controls<br>*N* (%)	*P*[a]
**All subjects**	1121	1216	
**Age, year**			0.501
**Range**	21–86	22–89	
**Mean**[b]	58.64 ± 11.31	58.97 ± 12.03	
**≤ 50**	232 (20.7)	270 (22.2)	
**51–60**	382 (34.1)	386 (31.8)	
**61–70**	338 (30.1)	378 (31.1)	
**> 70**	169 (15.1)	182 (14.9)	
**Sex**			0.326
**Females**	323 (28.8)	373 (30.7)	
**Males**	789 (71.2)	843 (69.3)	
**Smoking status**			< 0.001
**Never**	687(61.3)	619 (50.9)	
**Ever**	434 (38.7)	597 (49.1)	
**Drinking status**			0.008
**Never**	855 (76.3)	869 (71.5)	
**Ever**	266 (23.7)	347 (28.5)	
**Tumor site**[c]			
**GCA**	305 (27.2)	–	
**NGCA**	816 (72.8)	–	
**WHO classification**[d]			
**Low**	366 (32.6)	–	
**Medium**	542 (48.3)	–	
**High**	213 (19.0)	–	
**TNM stage**			
**I**	243 (21.7)	–	
**II**	245 (21.8)	–	
**III**	436 (38.9)	–	
**IV**	197 (17.6)	–	

[a]Two-side test for $\chi 2$ distributions between cases and controls.
[b]Data are presented as mean ± SD.
[c]GCA: gastric cardia adenocarcinoma, NGCA: non-gastric cardia adenocarcinoma.
[d]All patients were diagnosed as adenocarcinoma.
